# 人体内多环芳烃及其衍生物的代谢转化研究进展

**DOI:** 10.3724/SP.J.1123.2024.11030

**Published:** 2025-06-08

**Authors:** Jiankun QIAN, Runming HE, Ke FANG, Chenlong LI, Shan BAO, Wen GU, Song TANG

**Affiliations:** 1.中国疾病预防控制中心环境与人群健康重点实验室，中国疾病预防控制中心环境与健康相关产品安全所，北京 100021; 1. Key Laboratory of Environment and Population Health，National Institute of Environmental and Health-related Product Safety，Chinese Center for Disease Control and Prevention，Beijing 100021，China; 2.中国医科大学公共卫生学院，辽宁 沈阳 110122; 2. School of Public Health，China Medical University，Shenyang 110122，China; 3.山东大学齐鲁医学院公共卫生学院，山东 济南 250012; 3. School of Public Health，Cheeloo College of Medicine，Shandong University，Jinan 250012，China; 4.南京医科大学公共卫生学院全球健康中心，江苏 南京 211166; 4. Center for Global Health，School of Public Health，Nanjing Medical University，Nanjing 211166，China

**Keywords:** 多环芳烃, 衍生物, 代谢, 生物标志物, 人体生物监测, 综述, polycyclic aromatic hydrocarbons （PAHs）, derivatives, metabolism, biomarkers, human biomonitoring, review

## Abstract

多环芳烃（polycyclic aromatic hydrocarbons，PAHs）广泛存在于各类生产及生活环境中，因毒性高对公众健康构成了严重威胁。PAHs及其衍生物可通过多种途径进入人体，在细胞色素P450酶的催化作用下，生成Ⅰ相代谢产物，并进一步在Ⅱ相代谢阶段与谷胱甘肽、葡萄糖醛酸等物质结合，生成水溶性结合产物。不同代谢阶段的产物在人体内的分布与表现形式可作为PAHs暴露的重要标志物。然而，由于不同结构特征的PAHs在代谢机制及其产物种类方面存在显著差异，针对性细化研究策略对于基于生物监测的健康风险评估具有重要意义。系统开展PAHs及其衍生物代谢产物的时-量-效关系研究，明确PAHs及其衍生物的代谢产物类型及体内暴露水平，不仅有助于建立精准、高效、稳定的生物标志物谱库，还可为进一步开展人体生物监测提供科学依据和技术支撑。有鉴于此，本文系统梳理了母体PAHs及其硝基化、氧化和烷基化衍生物的主要代谢途径及产物类别，重点探讨了母体PAHs在人体内代谢转化的最新研究进展，旨在为揭示PAHs及其衍生物的暴露特征、健康风险评估和暴露溯源等研究提供科学参考。

## Abstract (en)

Polycyclic aromatic hydrocarbons （PAHs） are organic compounds produced primarily through the incomplete combustion of coal， petroleum， and other carbon-based materials. These compounds are environmentally ubiquitous and have attracted widespread attention because they are significantly biologically toxic and have far-reaching implications for public health and societal wellbeing. Consequently， developing a comprehensive understanding of how PAHs and their derivatives metabolically biotransform in the human body is critical for devising precise preventive strategies and targeted health interventions. PAHs and their derivatives metabolically transform *in vivo* in a complex process involving a broad variety of enzymes and pathways， and are usually divided into three distinct phases. Phase I encompasses oxidative， reductive， and hydrolytic reactions that are primarily catalyzed by cytochrome P450 （CYP） enzymes. These processes produce intermediates such as monohydroxyls， diols， diol-epoxides， and quinones， some of which （e.g.， diol-epoxides） form covalent DNA adducts， thereby contributing to their toxicities. Phase Ⅱ involves conjugation reactions， such as glucuronidation， sulfation， and glutathionylation， which enhance the water solubilities of the metabolites and facilitate their elimination. These detoxified metabolites are actively transported and excreted *via* bile or urine in phase Ⅲ， which effectively minimizes internal PAH exposure and prevents accumulation. Metabolites generated at various stages of PAH metabolism serve as crucial biomarkers for assessing human exposure levels. For example， urinary monohydroxy PAH metabolites （e.g.， 1-hydroxypyrene） have been widely adopted as reliable biomarkers for characterizing PAH exposure. However， owing to their structural diversity， PAHs metabolize via considerably different mechanisms to afford a variety of products， which highlights the need to differentiate individual PAHs and their derivatives in order to precisely assess exposure and evaluate nuanced health risks. Understanding the time-dose-effect relationships of PAH metabolites provides another major PAH-biomonitoring challenge. Investigating these dynamics is essential for revealing the cumulative and long-term health effects associated with exposure to multiple PAHs and their derivatives. Moreover， such studies provide scientific bases for formulating personalized and refined health-protection strategies. For instance， exploring how individual susceptibility， such as genetic polymorphisms in CYP enzymes or conjugation pathways， affects PAH metabolism is expected to significantly improve risk stratification and targeted interventions. PAH exposure is associated with significant health risks because they are associated with a range of diseases， including lung， pancreatic， and gastrointestinal cancers， as well as respiratory and cardiovascular diseases. The pervasive environmental presence of PAHs further complicates exposure scenarios， necessitating the comprehensive monitoring of various populations and environmental contexts. In addition to individual exposure， population-scale studies are expected to inform public health policies and regulatory actions aimed at reducing PAH exposure， particularly in vulnerable populations. This review concisely summarizes the metabolic pathways and product categories associated with four types of PAHs： parent， nitroxylated， oxidized， and alkylated. It emphasizes recent advances in our understanding of parent PAH metabolism in humans， focusing on their implications for exposure characterization， health risk assessment， source tracing， and regulatory decision-making. This paper aims to provide a scientific foundation for the advancement of human biomonitoring efforts and the development of evidence-based public health interventions tailored to reduce the burden of PAH exposure by addressing the complexities of PAH metabolism.

多环芳烃（polycyclic aromatic hydrocarbons，PAHs）是一类至少含有两个苯环，通过稠环形式连接而成的持久性有机污染物^［1‒3］^，具有较强的环境稳定性和潜在的生物毒性。根据其结构特征，可分为具有2或3个苯环的轻芳香烃（low-molecular-weight PAHs，LPAHs）和具有4个或更多苯环的重芳香烃（high-molecular-weight PAHs，HPAHs）^［4，5］^。在环境中，PAHs可通过光化学反应或微生物作用转化生成一系列衍生物^［6］^，如烷基化（alkylated-PAHs，APAHs）、氧化（oxygenated-PAHs，OPAHs）、硝基化（nitrated-PAHs，NPAHs）和卤代化多环芳烃（halogenated-PAHs，XPAHs）。这些衍生物的毒性效应往往显著高于母体PAHs^［7，8］^，对生态系统和人类健康构成更大的威胁。例如，2012年，1-硝基芘被国际癌症研究机构（International Agency for Research on Cancer，IARC）列为2A类致癌物，即对人类可能致癌，而芘仅被列为3类致癌物^［9］^；6-氯-苯并［*a*］芘对人L02肝细胞氧化应激效应显著高于苯并［*a*］芘，对细胞活力的抑制作用也更为明显^［10］^。

研究PAHs及其衍生物在机体内的代谢转化机制，不仅有助于理解其在生物体内的吸收、分布、代谢和排泄全过程，还为解析其复杂的代谢途径、关键酶系及重要代谢产物提供了科学依据，也为开发有效的防控策略和干预措施奠定坚实基础。尽管目前对苯并［*a*］芘一类PAHs研究已较为深入，针对其他类型PAHs的体内代谢种类鉴定及其代谢转化率仍亟待研究^［11］^。尤其是，不同暴露途径显著影响 PAHs 的代谢途径及其代谢产物。例如，大鼠研究表明，口服摄入2-硝基芴时，主要在肠道被代谢为2-氨基芴；而通过呼吸道吸入暴露则主要在肝脏被代谢为羟基化硝基芴^［12］^。这一发现表明，暴露途径直接决定了代谢转化的器官特异性和主要代谢产物类型。此外，不同遗传背景的生物体对PAHs及其衍生物的代谢存在差异^［13］^。例如，谷胱甘肽-*S*-转移酶T1（glutathione *S*-transferase，GSTT1）具有解毒作用，该基因的存在与否直接影响PAHs的代谢效率。基因*GSTT1*携带者尿1-羟基芘浓度比缺失者高1.2倍^［14］^。这一结果突显了代谢相关基因多态性在个体代谢差异中的重要作用，同时为精准风险评估提供了可能的分子标志。不同PAHs及其衍生物的代谢产物种类繁多，代谢转化路径错综复杂，增加了筛选主要代谢产物和关键代谢转化路径的难度。

综上，为深入了解PAHs及其衍生物在生物体内的代谢转化途径，确定不同代谢阶段产物的结构特征，系统阐明其在人体内的代谢转化规律，科学评估其暴露的健康风险并制定精准的防控与干预措施，本文总结评述了PAHs及其衍生物在生物体内的代谢途径、代谢产物及其特征。

## 1 PAHs体内代谢途径与代谢产物

PAHs在生物体内的转化过程包括3个阶段^［15］^：Ⅰ相代谢是氧化反应，PAHs通过氧化、还原和水解生成酚类、醌类和环氧二醇等物质，部分产物可与DNA或蛋白质结合形成加合物，引发肿瘤^［16，17］^。Ⅱ相代谢涉及结合反应，第Ⅰ阶段产物与谷胱甘肽、葡萄糖醛酸和硫酸等结合生成无毒水溶性物质，葡萄糖醛酸和硫酸结合物通过胆汁和尿液排出体外，谷胱甘肽结合物在肾脏中转化为巯基尿酸并随尿液排出^［18］^。Ⅱ相代谢的核心在于减少PAHs代谢产物的毒性和生物累积性，从而降低对机体的长期危害。Ⅲ相代谢是转运反应，利用ABC转运蛋白，通过腺嘌呤核苷三磷酸（adenosine triphosphate，ATP）水解产生的能量，逆浓度梯度将Ⅱ阶段生成的亲水性物质主动转运出细胞^［15，19-22］^。该阶段对于确保PAHs代谢物的及时排出、减少其在体内的滞留至关重要。

当前研究大多集中于常见的PAHs，其中苯并［*a*］芘代谢产物及转化途径已基本明确（图1）。然而，对于其他PAHs的研究相对局限，主要集中于二醇-环氧化物途径，而对于诸如自由基-正离子途径等其他代谢途径的研究仍显不足。同时，针对PAHs衍生物的代谢研究及其生物学效应的研究相对缺乏。图2系统总结了目前已发现的PAHs及其衍生物的体内代谢转化途径和代谢产物，并在表1中总结了已报道的PAHs及其衍生物的代谢特征和研究进展。

**图1:**
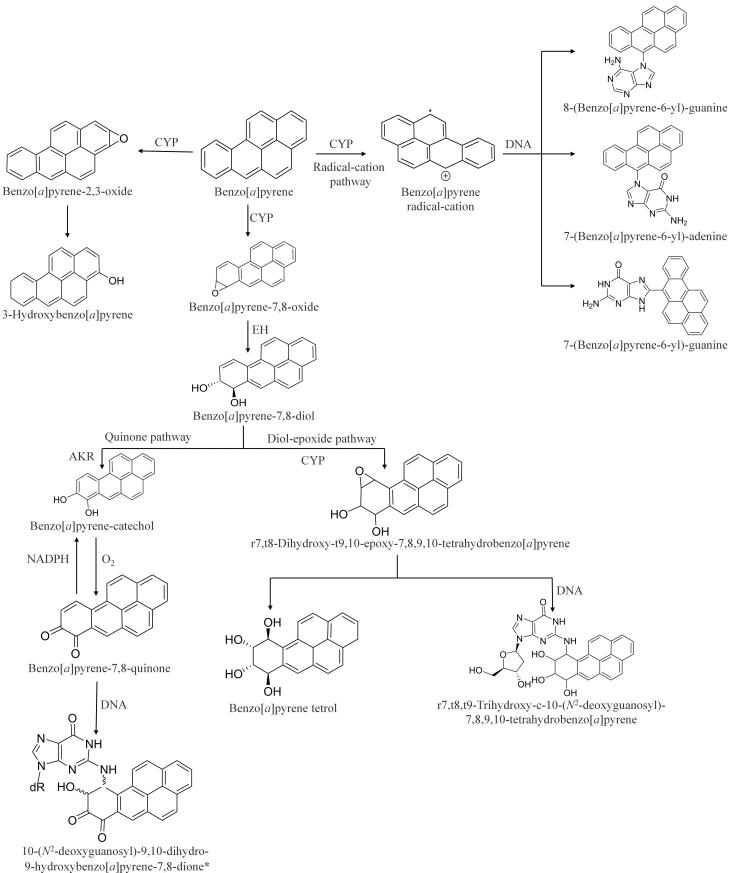
苯并［*a*］芘体内代谢途径

**图2:**
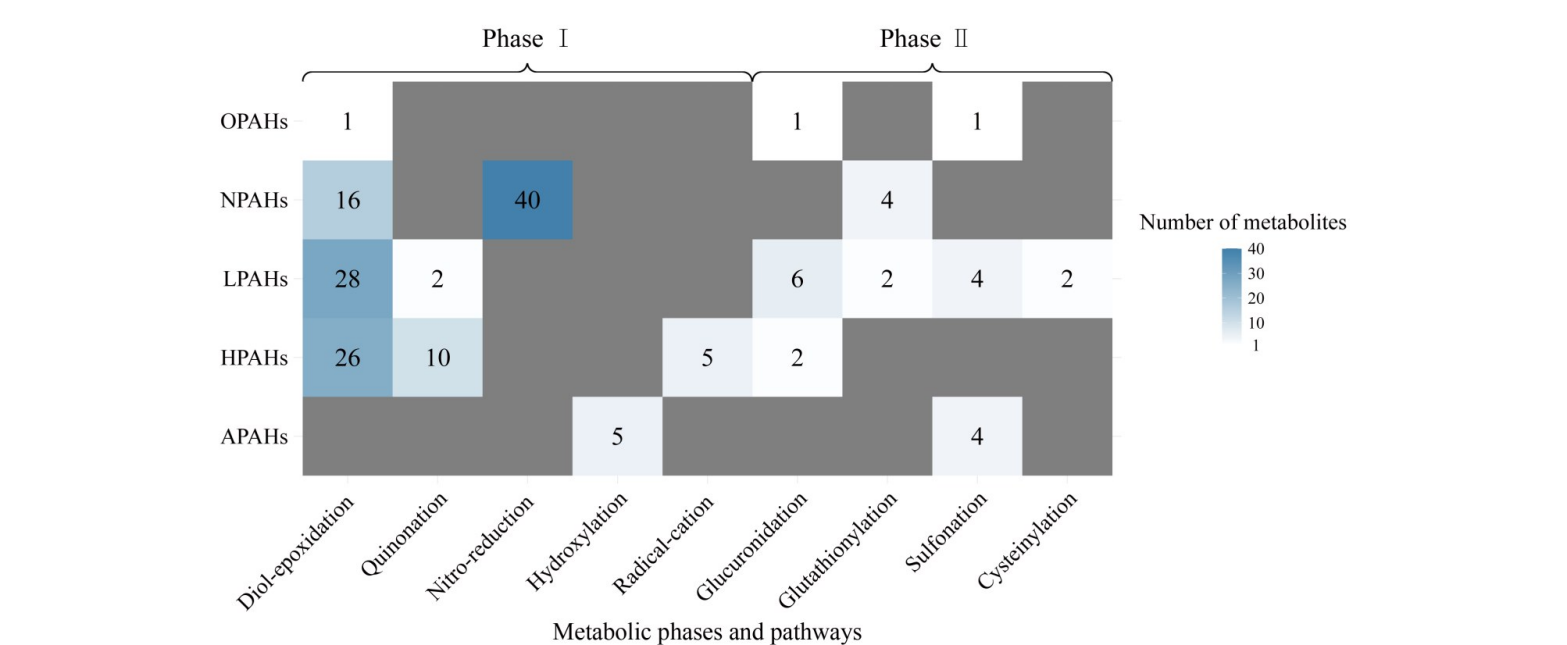
PAHs及其衍生物的代谢途径与代谢产物个数

**表 1:** PAHs及其衍生物的体内代谢研究现状

Compound	Categories	Phase Ⅰ metabolites	Phase Ⅱ metabolites	Refs.
Pyrene	HPAHs	1-hydroxypyrene， 1，6-dihydroxypyrene， 1，8-dihydroxypyrene， pyrene-1，6-quinone， pyrene-1，8-quinone	2 isomers of hydroxypyrene glucuronide	［^23^-^25^］
Benzo［*a*］anthracene	HPAHs	benzo［*a*］anthracene-3，4-dihydrodiol， *anti*-benzo［*a*］anthracene-3，4-dihydrodiol-1，2-epoxide， benzo［*a*］anthracene-3，4-catechol， benzo［*a*］anthracene-3，4-orthoquinone		［^26^，^27^］
Benzo［*a*］pyrene	HPAHs	benzo［*a*］pyrene radical-cation， 8-（benzo［*a*］pyrene-6-yl）-guanine， 7-（benzo［*a*］pyrene-6-yl）-adenine， 7-（benzo［*a*］pyrene-6-yl）-guanine， benzo［*a*］pyrene-2，3-oxide， 3-hydroxybenzo［*a*］pyrene， benzo［*a*］pyrene-7，8-oxide， benzo［*a*］pyrene-catechol， *trans*-7，8-dihydroxy-7，8-dihydrobenzo［*a*］pyrene， r7，t8-dihydroxy-t9，10-epoxy-7，8，9，10-tetrahydrobenzo［*a*］pyrene， r7，t8，t9-trihydroxy-c-10-（*N* ^2^-deoxyguanosyl）-7，8，9，10-tetrahydrobenzo［*a*］pyrene， benzo［*a*］pyrene tetrol， benzo［*a*］pyrene-7，8-quinone， 10-（*N* ^2^-deoxyguanosyl）-9，10-dihydro-9-hydroxybenzo［*a*］pyrene-7，8-dione^*^		［^28^-^32^］
Dibenzo［*a，h*］anthracene	HPAHs	3，4-arene oxide enantiomers of dibenz［*a，h*］anthracene， *trans-*dibenz［*a，h*］anthracene-3，4-dihydrodiol， dibenz［*a，h*］anthracene-3，4-diol-1，2-epoxide		［^33^］
Dibenzo［*def，p*］chrysene	HPAHs	dibenzo［*def，p*］chrysene radical cation， dibenzo［*def，p*］chrysene-11，12-epoxide， 2 isomers of dibenzo［*def，p*］chrysene-trans-11，12-dihydrodiol， 4 isomers of dibenzo［*def，p*］chrysene-*trans*-11，12-dihydrodiol-13，14-epoxide， 4 isomers of dibenzo［*def，p*］chrysene-11，12，13，14-tetrahydrotetraol， dibenzo［*def，p*］chrysene-11，12-catechol， dibenzo［*def，p*］chrysene-11，12-semiquinone， dibenzo［*def，p*］chrysene-11，12-quinone		［^34^］
Naphthalene	LPAHs	naphthalene-1，2-oxide， 1，2-dihydronaphthalene-1，2-diol， 1，2-dihydroxy-3，4-epoxy-1，2，3，4-tetrahydronaphthalene， 1-hydroxynaphthalene， 2-hydroxynaphthalene， naphthalene-1，2-diol， naphthalene-1，4-diol， 1，2-naphthalenedione， 1，4-naphthoquinone	1-hydroxy-2-glutathione-1，2-dihydronaphthalene， 1-glutathione-2-hydroxy-1，2-dihydronaphthalene， 2-hydroxynaphthaleneglucuronide， 1-hydroxynaphthaleneglucuronide， 2-hydroxynaphthalene-sulfate， 1-hydroxynaphthalene-sulfate	［^25^，^35^，^36^］
Fluorene	LPAHs	2-hydroxyfluorene， 3-hydroxyfluorene， 9-hydroxyfluorene	hydroxyfluorene sulfate， 3 isomers of hydroxyfluorene glucuronide	［^25^，^37^］
Phenanthrene	LPAHs	phenanthrene-1，2-oxide， 2-hydroxyphenanthrene， 1-hydroxyphenanthrene， 1，2-diol-phenanthrene， phenanthrene-（1*R*，2*S*）-diol-（3*S*，4*R*）-epoxide， （1*R*，2*S*，3*R*，4*S*）- phenanthrene-tetrol， phenanthrene-9，10-oxide， phenanthrene-9，10-diol， 9-hydroxyphenanthrene， 3，4-phenanthrene-oxide， 3-hydroxyphenanthrene， 4-hydroxyphenanthrene， phenanthrene-3，4-diol， phenanthrene-（3*S*，4*R*）-diol-（1*R*，2*S*）-epoxide， （1*R*，2*R*，3*S*，4*R*）-phenanthrene-tetrol	hydroxyphenanthrene-glucuronide， 2-hydroxyphenanthrene-sulfate， 2 isomers of phenanthrene-dihydrodiol-glycine	［^23^-^25^，^38^］
Anthracene	LPAHs	anthracene-l，2-oxide， anthracene-1，2-dihydrodiol		［^39^］
Fluoranthene	LPAHs	*anti*-2，3-dihydroxy-1，10b-epoxy-1，2，3-trihydrofluoranthene		［^40^］
1-Nitropyrene	NPAHs	1-nitropyrene-4，5-oxide， 1-nitropyrene-9，10-oxide， 1-nitropyrene-4，5-dihydrodiol， 1-nitropyrene-9，10-dihydrodiol， 1-nitrosopyrene， *N*-hydroxy-1-aminopyrene， 1-aminopyrene， *N*-acetyl-1-aminopyrene， 8-（deoxyguanosin-*N* ^2^-yl）-1-aminopyrene， （*N*-deoxyguanosin-8-yl）-1-aminopyrene	4-（glutathione-*S*-yl）-5-hydroxy-4，5-dihydro-1-nitropyrene， 5-（glutathione-*S*-yl）-4-hydroxy-4，5-dihydro-1-nitropyrene， 9-（glutathione-*S*-yl）-10-hydroxy-9，10-dihydro-1-nitropyrene， 10-（glutathione-*S*-yl）-9-hydroxy-9，10-dihydro-1-nitropyrene	［^41^-^44^］
1-Nitrobenzo［*a*］pyrene	NPAHs	1-nitrobenzo［*a*］pyrene-7，8-epoxide， 1-nitrobenzo［*a*］pyrene-7，8-diol， 1-nitro-*trans*-7，8-dihydroxy-9，10-epoxy-7，8，9，10-tetrahydrobenzo［*a*］pyrene， *N*-hydroxy-1-aminobenzo［*a*］pyrene， 1-aminobenzo［*a*］pyrene		［^45^］
1，6-Dinitropyrene	NPAHs	1-amino-6-nitropyrene， 1-nitro-6-nitrosopyrene， *N*-（deoxyguanosin-8-yl）-1-amino-nitropyrene		［^42^，^44^］
1，8-Dinitropyrene	NPAHs	1-nitroso-8-nitropyrene， *N*-hydroxy-1-amino-8-nitropyrene， 1-amino-8-nitropyrene， *N*-acetoxy-1-amino-8-nitropyrene， *N*-（deoxyguanosin-8-yl）-1-amino-8-nitropyrene		［^42^，^44^］
2-Nitrofluorene	NPAHs	*N*-hydroxy-2-aminofluorene， *N*-hydroxy-2-acetylaminofluorene， *N*-deoxyguanosin-8-yl-2-aminofluorene		［^46^］
3-Nitrofluoranthene	NPAHs	*N*-hydroxy-3-aminofluoranthene， *N*-（deoxyguanosin-8-yl）-3-aminofluoranthene		［^44^］
3-Nitrobenzanthrone	NPAHs	3-aminobenzanthrone， *N*-acetyl-*N*-hydroxy-3-aminobenzanthrone， *N*-acetoxy-*N*-acetyl-3-aminobenzanthrone， *N*-hydroxy-3-aminobenzanthrone， *N*-acetoxy arylamines， *N*-sulfooxy arylamines， electrophilic nitrenium ions， 8-（3-amino-7H-benz［*de*］anthracen-7-one-2-yl）-2′-deoxyguanosine， 8-（3-amino-7H-benz［*de*］anthracen-7-one-*N*-yl）-2′-deoxyguanosine， *N*_2_-（3-amino-7H-benz［*de*］anthracen-7-one-2-yl）-2′-deoxyguanosine		［^47^-^50^］
5-Nitroacenaphthene	NPAHs	1-hydroxy-5-nitroacenaphthene， 1-hydroxy-5-aminoacenaphthene， 1-keto-5-nitroacenaphthene， 1-keto-5-aminoacenaphthene		［^44^］
6-Nitrochrysene	NPAHs	*trans*-1，2-dihydro-1，2-dihydroxy-6-nitrochrysene， *trans*-1，2-dihydro-1，2-dihydroxy-6-aminochrysene， *trans*-1，2-dihydroxy-3，4-epoxy-1，2，3，4-tetrahydro-6-aminochrysene， 6-nitrosochrysene， *N*-hydroxy-6-aminochrysene， 5-（deoxyguanosin-*N* ^2^-yl）-6-aminochrysene， *N*-（deoxyguanosin-8-yl）-6-aminochrysene， *N*-（deoxyinosin-8-yl）-6-aminochrysene		［^44^，^51^-^53^］
7-Nitrobenzo［*a*］anthracene	NPAHs	7-nitrobenz［*a*］anthracene-*trans*-3，4-epoxide， 7-nitrobenz［*a*］anthracene-*trans*-8，9-epoxide， 7-nitrobenz［*a*］anthracene-*trans*-3，4-dihydrodiol， 7-nitrobenz［*a*］anthracene-*trans*-3，4-dihydrodiol-1，2-epoxide， 7-nitrobenz［*a*］anthracene-1，2，3，4-tetrahydrotetrol， 7-nitrobenz［*a*］anthracene-*trans*-8，9-dihydrodiol		［^44^］
1-Methylpyrene	APAHs	1-hydroxymethylpyrene， *N* ^6^-（1-methylpyrenyl）-2′-deoxyadenosine， *N* ^2^-（1-methylpyrenyl）-2′-deoxyguanosine	1-sulfoxymethylpyrene， cleavage of 1-sulfoxymethylpyrene to electrophilic positive carbon ions	［^54^-^56^］
7，12-Dimethylbenz［*a*］anthracene	APAHs	7-hydroxymethyl-12-methylbenz［*a*］anthracene， 7-methyl-12-hydroxymethylbenz［*a*］anthracene	7-methyl-12-sulfooxymethylbenz［*a*］anthracene， 7-sulfooxymethyl-12-methylbenz［*a*］anthracene	［^57^］
9，10-Phenanthrenequinone	OPAHs	9，10-dihydroxyphenanthrene	9，10-dihydroxyphenanthrene monoglucuronide， 9，10-dihydroxyphenanthrene monosulfonated	［^38^，^58^］

### 1.1 自由基-正离子途径

PAHs通过活性亲电试剂催化形成自由基正离子，这一单电子氧化在其代谢活化中具有重要作用。活性亲电试剂主要由细胞色素P450酶系（cytochrome P450， CYP）和过氧化物酶（如辣根过氧化物酶（horseradish peroxidase， HRP）和前列腺素H合成酶（prostaglandin H synthase， PHS））通过去除一个*π*电子产生^［59］^。苯并［*a*］芘在CYP催化下发生单电子氧化，在C6位生成自由基正离子，并与DNA结合形成加合物。通过腹腔注射^［60］^和皮肤接触暴露后，小鼠体内形成的DNA加合物包括8-（苯并［*a*］芘-6-基）-鸟嘌呤、7-（苯并［*a*］芘-6-基）-腺嘌呤和7-（苯并［*a*］芘-6-基）-鸟嘌呤，其中8-（苯并［*a*］芘-6-基）-鸟嘌呤占比最高（34%）^［61］^。由于DNA加合物形成与致癌性密切相关，8-（苯并［*a*］芘-6-基）-鸟嘌呤的高比例提示其在苯并［*a*］芘诱发的遗传毒性中发挥重要作用，需在毒性效应和健康风险评估中重点关注。

### 1.2 二醇-环氧化物途径

PAHs二醇-环氧化物途径是其生物体内活性代谢物生成的主要途径。首先，PAHs经CYP（如1A1、1A2、1B1）^［20］^催化生成环氧化物，继而通过环氧化物水解酶（epoxide hydrolase，EH）代谢为二羟基或二醇，最后被CYP氧化为二醇-环氧化物。二醇-环氧化物可与DNA结合形成加合物，或被进一步代谢为四醇类物质。以苯并［*a*］芘为例，在CYP1A1/1B1酶^［29］^的作用下，生成7，8-环氧化苯并［*a*］芘，随后被EH代谢生成苯并［*a*］芘-7，8-二醇，再进一步被CYP1A1/1B1代谢，形成反式-7，8-二羟基-9，10-环氧-7，8，9，10-四氢苯并［*a*］芘（BPDE），这是苯并［*a*］芘的最终致癌物^［30］^。BPDE与DNA结合形成r7，t8，t9-三羟基-c-10-（*N* ^2^-脱氧鸟苷）-7，8，9，10-四氢苯并［*a*］芘^［28，29］^，直接导致基因损伤和致癌效应。

### 1.3 醌途径

醌途径与二醇-环氧化物途径的代谢产物相似，主要区别在于生成二羟基或二醇后被醛酮还原酶（aldo-keto reductase，AKR）催化形成邻苯二酚，邻苯二酚随后被氧化为邻醌。例如，苯并［*a*］芘在CYP1A1/1B1和EH作用下生成苯并［*a*］芘-7，8-二醇，随后通过AKR1A1催化脱氢生成邻苯二酚，与O_2_进入氧化还原循环生产苯并［*a*］芘-7，8-二酮。与此同时，苯并［*a*］芘-7，8-二酮也可通过还原型辅酶Ⅱ（reduced nicotinamide adenine dinucleotide phosphate，NADPH）还原生成邻苯二酚^［32］^。体外研究表明，苯并［*a*］芘-7，8-二酮与DNA结合形成的10-（*N* ^2^-脱氧鸟苷）-9，10-二氢-9-羟基苯并［*a*］芘-7，8-二酮具有较强的结构稳定性^［31］^，这一特性不仅使其成为评估长期暴露水平的潜在生物标志物，还可用于反映长期暴露水平，同时为PAHs代谢产物的机制研究提供了关键线索。

### 1.4 硝基还原途径

该特异性代谢通路是NPAHs在生物体内发挥毒性和致癌作用的关键路径。硝基通过一系列顺序步骤，在CYP（1A1、1A2、1B1、3A4）、氧化酶（醛氧化酶、黄嘌呤氧化酶）和AKR等^［62］^酶的催化下转化为氨基。1-硝基芘首先通过去除2个电子还原生成1-亚硝基芘，进一步还原生成*N*-羟基-1-氨基芘，最终生成1-氨基芘。*N*-羟基-1-氨基芘可通过独立于乙酰化的氮离子与DNA形成加合物，或通过*N，O*-乙酰转移酶发生共轭反应，生成*N*-乙酰氧基-1-氨基芘，并与DNA加合。随后，N-O裂变可形成氮和碳离子互变异构体，分别形成*N*-（脱氧鸟苷-8-基）-1-氨基芘、8-（脱氧鸟苷-*N* ^2^-基）-1-氨基芘和6-（脱氧鸟苷-*N* ^2^-基）-1-氨基芘^［41-43］^（图3）。这些加合物不仅具有高毒性和高结构稳定性，还可对基因突变和DNA损伤起关键作用。

**图3:**
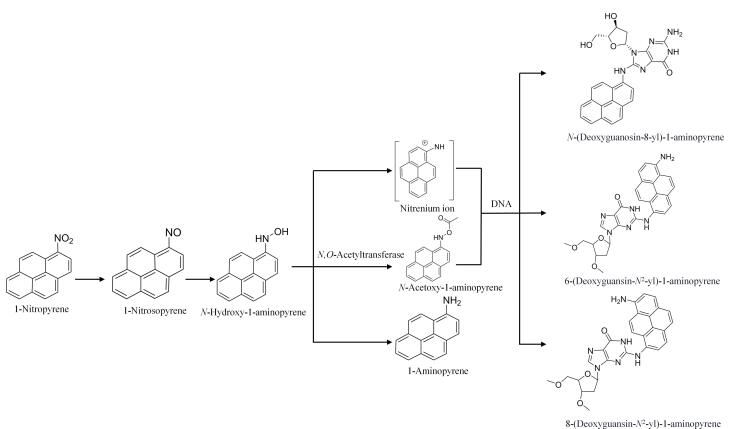
体内1-硝基芘硝基还原途径

### 1.5 羟基化和磺化途径

APAHs的体内代谢转化主要涉及羟基化和磺化途径。APAHs首先通过CYP（如CYP2F2）^［63］^生成羟基代谢物，再通过磺基转移酶（sulfotransferase，SULT）转化为磺基产物，与DNA结合形成加合物^［11］^。尽管磺化反应是Ⅱ阶段反应，但生成的许多硫酸酯能够与亲核试剂（如DNA和蛋白质）反应，增加致癌潜力^［64，65］^。1-甲基芘由于缺乏末端角位苯环，无法形成二醇-环氧化物^［54，66］^。因此，其代谢先由CYP2F2代谢生成1-羟甲基芘，再经SULT转化为1-甲磺基芘，后者与DNA结合形成*N* ^2^-（1-甲基芘）-2′-脱氧鸟苷和*N* ^6^-（1-甲基芘）-2′-脱氧腺苷^［56，67］^（图4）。这些加合物因其稳定性和与DNA关键位点的结合能力，成为评估APAHs致癌风险的重要指标。

**图4:**
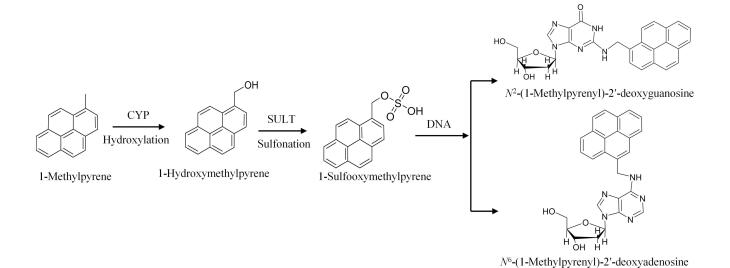
体内1-甲基芘羟基化和磺化途径

## 2 PAHs人体代谢研究及其生物监测应用

近年来，PAHs在生物体内代谢特征研究备受关注。深入探究其代谢产物和影响因素，有助于全面理解PAHs在生物体内的动态行为。当前，研究PAHs及其衍生物代谢的方法包括体外实验^［68］^和动物模型^［69，70］^。体外实验通常使用肝微粒体或细胞系模拟人体代谢过程，而动物实验则提供更为全面的体内代谢动力学数据，揭示代谢物的清除速率、半衰期等关键参数，从而阐明PAHs的生物体内代谢机制。

### 2.1 动力学分析模型在PAHs体内代谢研究中的应用

为更准确地模拟PAHs在人体内的代谢行为，既往研究大多利用动力学数据分析模型，包括非线性混合效应模型、区室模型和生理药代动力学模型。这些模型通过结合生理学参数与生物样本（如尿液、血液和粪便）中的实测数值，能够有效预测不同暴露情境下PAHs的体内分布浓度。其中，生理药代动力学模型因其适用性广泛，已成为环境暴露研究中的关键工具之一，并利用体内、外实验对参数进行了优化。例如，Kim等^［71］^通过室内暴露实验优化了人皮肤对萘的吸收参数，包括角质层渗透系数和脂肪血液分配系数，推动了生理药代动力学模型的结构优化。Deng等^［72］^通过构建生理药代动力学模型，模拟不同年龄人群对苯并［*a*］芘及其代谢产物3-羟基苯并［*a*］芘的药代动力学过程，揭示了年龄差异对暴露风险的显著影响。表2和表3分别总结了苯并［*a*］芘、3-羟基苯并［*a*］芘^［72］^、萘^［71］^和二苯并［*def*，*p*］䓛^［73］^的人体生理药代动力学模型参数。然而，人和动物之间存在显著的生理差异，以及不同化合物理化特性差异，导致许多模型参数无法直接应用于不同物种。此外，PAHs及其衍生物的模型构建还面临以下挑战：（1）PAHs与其他污染物的混合暴露可能加剧其毒性，增加健康风险。（2）相对分子质量较大的PAHs更易附着于颗粒物，其进入机体的机制不同于气态物。（3）虽然PAHs多以较低剂量进入人体，短期暴露难以显现毒性，但长期暴露对人类健康构成巨大威胁。因此，亟需开发更多适用于人体暴露不同PAHs及其衍生物的动力学数据分析模型。

**表 2:** 不同年龄段的生理参数值^［71‒73］^

Parameter	Newborn	1 year	5 years	10 years	15 years	Adult （30 years）
Body weight （kg）	3.5	10.0	19.0	32	56	73
Alveolar ventilation rate， *Q*_P _（L/h）	90.0	150.0	240.0	310	420	450
Cardiac output， *Q*_C _（L/h）	35.4	90.0	192.0	264	354	366
Blood volume， *V*_B _（L）	0.3	0.5	1.4	2.4	4.5	5.3
Blood ﬂow （L/h）						
Skin， *Q*_SK_	1.80	3.60	10.20	15.0	18.9	19.50
Adipose， *Q*_AD_	1.80	3.60	10.26	15.0	18.9	19.50
Kidney， *Q*_K_	6.60	13.80	34.62	51.2	80.1	79.50
Liver， *Q*_L_	2.34	4.68	13.26	19.5	24.5	25.38
Rapidly perfusion tissues， *Q*_RP_	15.84	44.52	86.52	114.1	143.2	151.32
Slowly perfusion tissues， *Q*_SP_	7.02	22.68	37.14	49.2	68.4	70.80
Tissue volumes （L）						
Skin， *V*_SK_	0.18	0.35	0.57	0.82	2.000	3.300
Adipose， *V*_AD_	0.93	3.80	5.50	8.60	12.00	18.20
Kidney， *V*_K_	0.03	0.07	0.11	0.18	0.250	0.310
Lung， *V*_LU_	0.03	0.08	0.13	0.21	0.330	0.500
Liver， *V*_L_	0.13	0.33	0.57	0.83	1.300	1.800
Slowly perfusion tissues， *V*_SP_	1.25	2.59	6.74	12.15	23.744	27.702

**表 3:** 所有年龄段不同组织/器官的人体生理药代动力学模型参数^［71‒73］^

Parameter	BaP	3-BaP	NAP	DBC
Partition coefficients				
Blood/air， *P*_ab_	590.00	-	10.3	-
Lung， *P*_lu_	1.37	2.92	-	1.13
Adipose， *P*_ad_	142.00	1.42	25.6	158.33
Skin， *P*_sk_	5.31	0.80	2.8	-
Kidney， *P*_ki_	8.98	40.4	-	-
Liver， *P*_li_	8.39	1.83	-	9.38
Rapid perfusion， *P*_ra_	12.47	3.35	-	10.37
Slow perfusion， *P*_sl_	7.36	0.56	-	7.49
Metabolic constants				
Maximum metabolic rate per kilogram of tissue， *V*_max_ （µmol/h）	705.8	-	-	-
Michaelis constant， *K*_m_ （µmol/L）	5.5	-	-	-
*V*_max_/*K*_m _（L/h）	-	37.13	698	-
Fraction of hydroxy metabolites， *f*	0.185	-	-	-
Elimination rates （1/h）				
Biliary， *K*_lgi_ （from liver to gastrointestinal tract）	0.042	0.042	-	-
*K*_gil_ （from gastrointestinal tract to liver）	-	0.007	-	-
Urine， *K*_kb_ （from kidney to bladder）	7.500	7.500	-	-
*K*_u_ （from bladder to urine）	0.009	0.009	-	-
Fecal， *K*_f_	0.334	0.173	-	-
Skin permeation parameters				
Thickness of the stratum corneum， *T*_c_ （µm）	-	-	10	-
Total body surface areag， *SURFA* （cm^2^）	-	-	19238	-
Permeability coefficient for stratum corneum， *K*_ps_ （cm/h）	-	-	6.8×10^-5^	-
Permeability coefficient for viable epidermis， *K*_pv_ （cm/h）	-	-	3.0×10^-3^	-

-： not given in the reference； BaP： benzo［*a*］pyrene； 3-BaP： 3-hydroxybenzo［*a*］pyrene； NAP： naphthalene； DBC： dibenzo［*def，p*］chrysene.

### 2.2 PAHs体内代谢特征

通过筛查人体生物样本中的相关代谢产物，可有效延长暴露检测的时间窗口，提供机体摄入情况的确凿证据。因此，PAHs及其衍生物的代谢产物被认为是良好的候选暴露生物标志物。近年来，PAHs人体代谢研究取得了显著进展，主要集中在代谢动力学模型优化、代谢特征解析^［74］^以及代谢物作为生物标志物的应用等方面。Misra等^［75］^利用高分辨非靶向代谢组学和靶向蛋白质组学对皮肤样本进行分析，发现污染较严重地区人体内氨基酸和脂肪酸代谢通路的富集与PAHs显著相关。Wu等^［76］^利用高分辨非靶向代谢组学和KEGG通路分析指出地下停车场工人尿液中未代谢的HPAHs通过缺氧诱导因子1（hypoxia-inducible factor 1，HIF-1）信号通路诱导糖代谢紊乱，进而诱发糖尿病。此外，He等^［77］^指出，尿中的氨基PAHs可作为NPAHs的暴露生物标志物，而Lin等^［78］^表明，尿羧酸代谢物可能适用于APAHs的暴露生物标志物。同时，尿中1-羟基芘已被广泛作为PAHs内暴露的生物标志物^［79］^。这些研究表明，针对不同类型PAHs的丰度和结构特征，有必要选择不同的代谢产物作为暴露生物标志物。同时，基因多态性对PAHs体内代谢的影响也成为研究热点，基因多态性是影响个体对PAHs代谢能力的重要因素，尤其是在代谢酶活性和代谢产物生成方面。酶的遗传变异可能导致个体间代谢效率的显著差异，进而改变动力学模型的预测准确性。Guo等^［80］^利用单核苷酸多态性位点（single nucleotide polymorphisms sites， SNPs）标记方法对苯并［*a*］芘代谢酶进行分型，发现携带*CYP1A1 TT*基因型的个体，其BPDE-白蛋白加合物浓度是*CYP1A1 CC*基因型个体的0.94倍。此外，Wang等^［81］^研究指出，*CYP1A1 AG*基因型与*CYP2E1 CC*基因型联合作用与食管癌的易感性显著相关。然而，目前多数研究仅关注与PAHs代谢相关的少数基因，并局限于探讨某一或少数几个基因位点的影响，未能全面揭示基因多态性在PAHs代谢差异中的作用机制。这种局限性提示未来研究应进一步扩大基因筛查范围，系统分析基因与代谢表型的关联。

Gideon等^［82］^通过研究成年吸烟者戒烟10天后的尿液单羟基代谢物的浓度变化，发现2-羟基萘的变化程度最为显著（第1天与第10天的尿浓度平均比值为21.6），而2-羟基菲的变化最小（平均比值为3.4），提示2-羟基萘代谢速度最快，2-羟基菲代谢速度最慢。Madeen等^［83］^通过人体口服微剂量^14^C-二苯并［*def，p*］䓛实验，发现血浆中的主要代谢物为^14^C-二苯并［*def，p*］䓛-11，12-二醇，尿液中的主要代谢物为^14^C-（+/‒）-二苯并［*def，p*］䓛-四醇，其中88.7%±6.5%的代谢物以结合形态存在。Gaudreau等^［84］^指出，尿液样本中单羟基代谢物结合态占比大于91%，提示Ⅱ相代谢物可能是更为理想的暴露生物标志物。表4总结了PAHs在人群体内的代谢特征。目前，关于加合物与Ⅱ相代谢物的研究相对较少。以上研究均提示Ⅱ相代谢物可能在体内具有更高的转化率，且对暴露水平的反映更加全面。因此，亟需加强对Ⅱ相代谢物的靶向检测分析，通过选择更加敏感的生物标志物，以更准确地评估PAHs人体内暴露水平和健康风险。

**表 4:** PAHs在人体内的代谢特征

Subjects	Age/years	Exposure route	Exposure substances	Kinetics data analysis methods	Sample type	Metabolic characterization	Ref.
9 adults	>18	ingestion	NAP， FLU， PHEN， PYR， and FRT	nonlinear mixed- effects model	urine	within 24 h； The parent PAHs， naphthalene， fluorene， phenanthrene， fluoranthene， and pyrene made up 0， 14%， 42%， 100%， and 56%， respectively， of the total PAH+hydroxy-PAH concentration measured in urine.	［^85^］
9 adults	23-61	ingestion	NAP， FLU， PHEN， and PYR	nonlinear mixed- effects model	urine	Mean percentage of PYR excreted as 1-PYR in urine over 24 h was 6.8% （range 4.5%‒14.6%）. For NAP， FLU， and PHEN， the mean percentages of their excreted hydroxylated metabolites （sum of the metabolites from the same PAH） were 182% （99%‒248%）， 60% （30%‒73%）， and 11% （7.5%‒16%）， respectively.	［^86^］
9 adults	20-65	ingestion	^14^C-DBC	non-compartmental model	urine	Total urinary excretion of ^14^C-DBC at 72 h was 1.24%±0.49% of the total oral dose.	［^87^］
6 adults	20-65	ingestion	^14^C-DBC	pharmacokinetic model	urine and plasma	^14^C-（+/‒）-DBC-11，12-diol was the major metabolite in plasma. ^14^C-（+/‒）-DBC-tetrol was the major metabolite in urine， of which 88.7%±6.5% was present in conjugated form.	［^83^］
7 adults	26-65	ingestion	^14^C-BaP	non-compartmental model and compartmental model	plasma	The metabolites with the highest yield were BaP-tetrols （specific stereoisomers unknown） and dihydrodiols （7，8- and 9，10- with perhaps some 4，5-dihydrodiol）.	［^88^］
8 adults	26-41	ingestion	NAP-d_8_， FLU-d_10_， PHEN-d_10_， and PYR-d_10_	non-compartmental model	urine	The means of 72 h fractional urinary excretion for each metabolite are as follows： 1.14%， 0.63%， 8.24%， 1.03%， 0.84%， 0.72%， 1.07%， 0.07%， 0.58%， and 11.3% for 1-， 2-NAP-d_7_， 2-， 3-FLU-d_9_， 1-， 2-， 3-， 4-， 9-PHEN-d_9_， 1-PYR-d_9_， respectively.	［^89^］
6 adults	20-65	ingestion	^14^C-DBC	PBPK model	urine and plasma	A smaller proportion of DBC gets eliminated via Phase I than Phase Ⅱ route （54% vs. 30%）.	［^73^］
Human liver microsomes	-	*in vitro*	BaP and DBC	PBPK model	-	The intrinsic clearance of BaP is nearly five-fold faster than that of DBC.	［^90^］
Human liver microsomes	-	*in vitro*	DBC	linear regression model and Michaelis-Menten equation	-	The metabolism rate of phase Ⅰ DBC-11，12-diol was 1.7-fold higher than that of DBC.	［^91^］

FLU： fluorene； PHEN： phenanthrene； PYR： pyrene； FRT： fluoranthene； 1-PYR： 1-hydroxypyrene； 1-NAP： 1-hydroxynaphthalene； 2-NAP： 2-hydroxynaphthalene； 2-FLU： 2-hydroxyfluorene； 3-FLU： 3-hydroxyfluorene； 1-PHEN： 1-hydroxyphenanthrene； 2-PHEN： 2-hydroxyphenanthrene； 3-PHEN： 3-hydroxyphenanthrene； 4-PHEN： 4-hydroxyphenanthrene； 9-PHEN： 9-hydroxyphenanthrene； PBPK： physiologically based pharmacokinetic.

### 2.3 PAHs代谢物在人体生物监测中的应用

人体生物监测（human biomonitoring，HBM）作为评估个体暴露于环境污染物的金标准，通过检测尿液、血液等生物样本中PAHs及其代谢产物，能够实时、准确反映个体PAHs的暴露水平。将PAHs代谢特征与人体生物监测深度融合，不仅可以更精确地量化个体暴露，还可动态评估潜在的健康风险。中国^［92］^、美国^［93］^、加拿大^［94］^、韩国^［95］^和欧洲^［96］^等各国家和地区生物监测项目中普遍选择尿液中单羟基代谢物（如1-羟基萘、2-羟基芴和1-羟基芘等）作为PAHs暴露的标志物。然而，研究表明1-羟基芘的代谢半衰期因暴露途径不同而存在显著差异，且普遍较短：吸入途径为6～35 h，摄入途径为4.4～12 h，皮肤接触途径则为11.5～15 h；此外，尿中单羟基代谢物浓度周内变异性为41%～60%，日内变异性为60%～84%^［97］^。Klotz等^［98］^评估了多种萘代谢物（1-羟基萘、2-羟基萘、1，2-二羟基萘、1-萘巯基巯基酸和2-萘巯基巯基酸）的适用性，发现1，2-二羟基萘最具有代表性。而Lin等^［99］^指出头发中单羟基代谢物比尿液中的更适合用于暴露评估，但二者均难以反映长期暴露。相比之下，DNA 或蛋白质加合物可提供长期暴露估计值。DNA加合物半衰期约为9~13周^［100］^，白蛋白加合物半衰期约为20天，血红蛋白加合物半衰期约为120天^［101，102］^。这表明，单纯依赖尿液中单羟基代谢物进行监测存在一定局限性，需要结合稳定性更强、更具代表性、检出率高的标志物以提升评估准确性。以上研究不仅揭示了不同代谢物在PAHs暴露监测中的优缺点，还为优化人体生物监测策略提供了坚实的科学依据，为更全面的环境暴露风险评估奠定了重要基础。

## 3 展望

PAHs广泛存在于环境中，其复杂多样的体内代谢途径会产生多种中间代谢产物和最终产物，为体内代谢研究带来严峻挑战。本综述系统梳理了母体PAHs及其硝基化、氧化和烷基化3种PAHs衍生物的主要代谢途径及产物类别，重点探讨了苯并［*a*］芘一类的母体PAHs在人体内代谢转化的最新研究进展，并指出当前世界主要国家和地区人体生物监测中的标志物均难以反映PAHs长期暴露。

现有研究大多聚焦于单羟基代谢物，而对于Ⅰ阶段其他中间代谢产物（如醌类、二醇类）和Ⅱ阶段代谢产物数据相对缺乏。此外，单羟基代谢物无法准确追溯原型物质来源，也不能反映长期暴露，其代谢物谱图还受取代基位置、个体代谢酶表型差异和代谢相互作用等因素的影响。当前尚未形成对PAHs及其衍生物的代谢途径和产物特征的全面而系统的科学认识。第一，PAHs衍生物虽然在环境中的浓度可能低于其对应的母体PAHs，但往往具有更强的持久性和生物累积性。为阐明PAHs衍生物的体内代谢特征，须利用基因敲除动物模型、人工智能和机器学习等先进技术方法，建立系统且精准的体内代谢转化研究体系，揭示其体内代谢通路和特征。同时，优化动力学数据分析模型，结合体外实验验证结果，为制定人群防控政策提供证据。其次，现有国家和地区的人体生物监测项目中PAHs指标均为尿单羟基代谢物，其不适合反映人体长期暴露的水平。为筛选长期暴露的生物标志物，应重点关注DNA和蛋白质加合物，因其可直接反映长期暴露水平及累积效应。结合Ⅰ相和Ⅱ相代谢产物进行综合分析，不仅能提高暴露评估的稳定性和准确性，还能推动非靶向筛查方法的标准化，利用高分辨率质谱、核磁共振等技术提高代谢物的识别效率，同时结合主成分分析和通路分析，确认关键代谢产物，为新型暴露生物标志物的检测方法建立奠定基础。第三，环境因素和遗传因素复杂多变，未来应探索遗传和环境的交互效应如何影响个体对PAHs的代谢和敏感性。将全基因组关联研究与多组学技术相结合，并引入环境因子与遗传因子的交互分析，探索基因-环境如何共同调控PAHs体内代谢。通过结合遗传学和环境数据，识别高风险人群，制定个性化的风险评估和预防策略，从而提升公众健康政策的科学性与针对性，为高风险人群提供精准干预的技术策略。
